# Post Traumatic Growth as a way of mastering COViD-19 Peritraumatic Distress Inde[ (in Russian sample)

**DOI:** 10.1192/j.eurpsy.2022.1328

**Published:** 2022-09-01

**Authors:** O. Kvasova, M. Magomed-Eminov, O. Savina, E. Karacheva, O. Magomed-Eminova

**Affiliations:** 1 Lomonosov Moscow State University, Psychological Helping And Resocialization, Moscow, Russian Federation; 2 Moscow State University, Psychological Helping And Resocialization, Moscow, Russian Federation; 3 Lomonosov Moscow State University, Psychology, Moscow, Russian Federation; 4 Moscow State University, Psychology Department, Moscow, Russian Federation; 5 Lomonosov Moscow State University, Department Of Psychological Help And Resocialization, Moscow, Russian Federation

**Keywords:** Covid-1, Covid-19, Peritraumatic Distress Index, coping, personality work, Peritraumatic Distress Index, coping

## Abstract

**Introduction:**

COVID-19 pandemic reality raise multiple problems that need effective ways of coping. Not only for people experienced contracting COVID-19 but those who did not the positive ways of coping are important way to overcome distress associated with COVID-19. Post-traumatic Growth of personality may be effective coping factor. To test this hypothesis we used several instruments: one of them - Peritraumatic Distress Index (CPDI) for assessing the level of distress specific to Covid-19

**Objectives:**

463 participants (including 66 patients in COVID-19 clinics)

**Methods:**

Russian version of Covid-19 Peritraumatic Distress Index (CPDI) validated in Psychological Helping and resocialization Department Moscow State University; Impact of Event Scale (Horowitz), Post-Traumatic Growth Inventory – PTGI (Tadeshi & Calhoun) adapted by M. Magomed-Eminov

**Results:**

Russian version of CPDI has high reliability-consistency (Cronbach’s α -0.87). We obtained from our data: significant correlation between CPDI and PTG for people experienced COVID-19 contamination. Content analysis of narratives and incomplete sentences showed: those who had higher scores on PTG and CPDI have differences in personal meaning of their traumatic experience.

**Conclusions:**

CPDI is presented in research as brief effective tool to identify COVID-19 related distress and plan helping strategies and psychiatric interventions for various people suffering by continuing pandemic crisis. Correlation between CPDI and COVID-19 contamination: could suggest more severe distress is associated with higher PTG. And PTG could be considered as positive factor coping with distress. We suggest cultural-activity approach to personality work with stressful experience of individual to confront distress, existential evaluation of life situation taking into account also resilience, growth .

**Disclosure:**

No significant relationships.

